# G-CSF/anti-G-CSF antibody complexes drive the potent recovery and expansion of CD11b^+^Gr-1^+^ myeloid cells without compromising CD8^+^ T cell immune responses

**DOI:** 10.1186/1756-8722-6-75

**Published:** 2013-10-01

**Authors:** Mark P Rubinstein, Mohamed L Salem, Andrew L Doedens, Caitlin J Moore, Cody Chiuzan, Guillermo L Rivell, David J Cole, Ananda W Goldrath

**Affiliations:** 1 Department of Biological Sciences, The University of California, San Diego, La Jolla, CA 92093, USA; 2 Department of Surgery, Medical University of South Carolina, Charleston, SC 29403, USA; 3 Department of Microbiology & Immunology, Medical University of South Carolina, Charleston, 86 Jonathan Lucas Street, HO506, SC 29403, USA; 4 Zoology Department, Faculty of Science, Tanta University, Tanta, Egypt; 5 Department of Public Health Sciences, Medical University of South Carolina, Charleston, SC 29425, USA

**Keywords:** G-CSF, Myeloid cells, Antibody/cytokine complexes, Pegylation, Neutrophils, Neupogen, Neulasta, Protein therapeutics

## Abstract

**Background:**

Administration of recombinant G-CSF following cytoreductive therapy enhances the recovery of myeloid cells, minimizing the risk of opportunistic infection. Free G-CSF, however, is expensive, exhibits a short half-life, and has poor biological activity *in vivo*.

**Methods:**

We evaluated whether the biological activity of G-CSF could be improved by pre-association with anti-G-CSF mAb prior to injection into mice.

**Results:**

We find that the efficacy of G-CSF therapy can be enhanced more than 100-fold by pre-association of G-CSF with an anti-G-CSF monoclonal antibody (mAb). Compared with G-CSF alone, administration of G-CSF/anti-G-CSF mAb complexes induced the potent expansion of CD11b^+^Gr-1^+^ myeloid cells in mice with or without concomitant cytoreductive treatment including radiation or chemotherapy. Despite driving the dramatic expansion of myeloid cells, *in vivo* antigen-specific CD8^+^ T cell immune responses were not compromised. Furthermore, injection of G-CSF/anti-G-CSF mAb complexes heightened protective immunity to bacterial infection. As a measure of clinical value, we also found that antibody complexes improved G-CSF biological activity much more significantly than pegylation.

**Conclusions:**

Our findings provide the first evidence that antibody cytokine complexes can effectively expand myeloid cells, and furthermore, that G-CSF/anti-G-CSF mAb complexes may provide an improved method for the administration of recombinant G-CSF.

## Background

The advent of recombinant G-CSF and related factors able to induce myeloid cell expansion have rapidly revolutionized the treatment of cancer and other diseases [1-5]. First cloned in 1986, G-CSF was FDA approved five years later as an adjuvant to overcome neutropenia, the dose-limiting toxicity of many cancer regimens. Consistent with its clinical use [6], administration of G-CSF induces multiple biological effects in mice, most notably, the expansion and activation of neutrophils and their progenitors [7-13]. It is the accelerated recovery of these cells that is thought to help reduce the risk of infection and other complications during many forms of cancer treatment. More recently, it was found that administration of G-CSF can also facilitate mobilization of hematopoietic progenitor cells to the peripheral blood. These mobilized progenitor cells are not only more easily accessible clinically than traditional bone marrow donor cells but more effective at inducing myeloid cell recovery following transfer to immunosuppressed patients [14,15]. Currently there are multiple indications for the administration of G-CSF including both the induction of myeloid cell recovery during iatrogenic or disease-related myelosuppression and the mobilization of progenitor cells in individuals donating peripheral blood stem cells for transplantation. G-CSF-related drugs represent one of the most important and widely used protein therapeutics in patients.

Despite the potency of G-CSF therapy, there are significant clinical limitations. Foremost, the half-life of recombinant G-CSF is relatively short (3.5 hours) [2,16]. This rapid loss of G-CSF *in vivo* means that the biological activity of administered G-CSF is transient and patients must receive repeated dosing to achieve a durable myeloid cell recovery. As a means of prolonging the half-life of G-CSF and improving its biological activity *in vivo*, it was found that, as with other cytokines [17,18], pegylation of G-CSF resulted in improved pharmokinectics and enhanced biological activity [2,19]. Thus, pegylated G-CSF has a half-life estimated at over 15 hours [16,20]. This effect is likely due to diminished renal clearance, as bilateral nephrectomy of rats decreases the clearance of free G-CSF but not pegylated G-CSF [21]. Because pegylated G-CSF can be safely administered with reduced dosing requirements, it was FDA-approved in 2002 [2], and it is now becoming the drug of choice where G-CSF is indicated. Despite these advances, there remain significant limitations with pegylated G-CSF [22-28]. It is difficult to manufacture and costly to administer large amounts of biologically active cytokine, and pegylation per se is known to reduce the *in vitro* activity of many cytokines. Furthermore, recent findings suggest the existence of anti-PEG (polyethylene glycol) antibodies in up to 25% of healthy individuals [27-29]. While the clinical significance of such antibodies in the administration of pegylated G-CSF is not known, anti-PEG antibodies have been documented to neutralize the activity PEG-uricase and PEG-asparaginase in human patients [25-27], and could provide an explanation for patients who fail to effectively respond to the administration of other pegylated protein therapeutics.

An alternative method for increasing the biological activity of a cytokine *in vivo* is pre-association with a cytokine-specific monoclonal antibody or a soluble receptor prior to injection. Thus, pre-association of IL-2, IL-3, IL-4, IL-6, IL-7, IFNα, or TNFα with specific cytokine-specific monoclonal antibodies dramatically improves biological activity *in vivo*[30-32]. Similarly, pre-association with soluble receptors can greatly improve the biological activity of IL-4, IL-6, and IFNα [32,33]. While these findings demonstrated that cytokine complexes could induce the activation and expansion of multiple cell types including B cells and mast cells, we and others have found that cytokine complexes, including with IL-15, could also be used to facilitate the expansion of T cells and natural killer cells [34-39].

Although cytokine complexes can effectively drive the expansion and activation of a wide range of cell populations *in vivo*, it is not clear whether cytokine complexes can universally act on all types of potential target cells [31]. The localization of cytokine complexes *in vivo* might favor their activity on some cell populations but not others. Alternatively, some cytokines may be inherently more amenable to enhanced activity when administered as a cytokine complex due to the mechanism by which they induce receptor signaling. Finally, it is possible that accessory cells necessary to facilitate the mechanism by which cytokine complexes act are not present after cytoreductive therapy. The latter would represent an obstacle limiting the use of cytokine complexes in many types of cancer therapy. Our purpose was to address these issues by the evaluation of cytokine-antibody complexes composed of G-CSF and anti-G-CSF mAb. Upon association, we find that these cytokine complexes are potent stimulators of neutrophils, a G-CSF-responsive cell type not previously shown to be responsive to cytokine complexes. Furthermore, our data demonstrate that these G-CSF/anti-G-CSF mAb complexes facilitate myeloid cell recovery after cytoreductive therapy without inducing a suppressive environment that compromises antigen-specific CD8^+^ T cell responses. These findings suggest a novel strategy for enhancing the clinical utility of recombinant G-CSF.

## Results

### Pre-association of G-CSF with anti-G-CSF mAb leads to greatly enhanced biological activity

**Figure 1:**
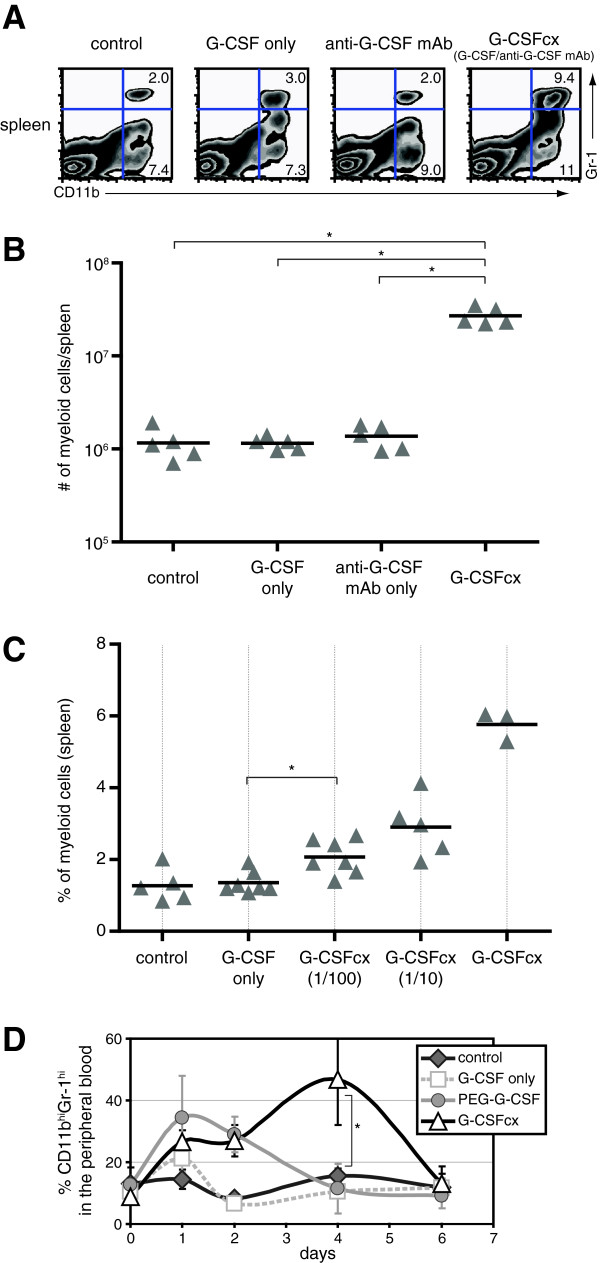
**G**-**CSF**/**anti**-**G**-**CSF mAb complexes exhibit enhanced biological activity in comparison with G**-**CSF. (A)** B6 mice were injected i.p. once with G-CSF/anti-G-CSF mAb complexes (0.5 μg G-CSF plus 2.5 μg anti-G-CSF mAb), cytokine alone (0.5 μg G-CSF), antibody alone (2.5 μg anti-G-CSF mAb), or vehicle alone. CD11b^+^Gr-1^+^ myeloid cells were identified by FACS analysis of spleens cells at 48 hours. **(B)** B6 mice were injected i.p. three times (on days 1, 3, and 5) with G-CSF/anti-G-CSF mAb complexes (1.5 μg G-CSF plus 7.5 μg anti-G-CSF mAb) or controls. Spleens were harvested on day 7. Shown are the number of CD11b^+^Gr-1^+^ myeloid cells identified by FACS. Each triangle represents an individual mouse and the bar indicates the mean. *P-values ≤ 0.05 were generated by Wilcoxon Rank-Sum test. **(C)** B6 mice were injected once with G-CSF only (1.5 μg) or with G-CSF/anti-G-CSF mAb complexes (1.5 μg G-CSF plus 7.5 μg anti-G-CSF mAb). For the 1/10 condition, mice were injected with 0.15 μg G-CSF plus 0.75 μg anti-G-CSF mAb, and for the 1/100 condition, an additional 10-fold dose reduction. The percent of CD11b^+^Gr-1^+^ myeloid cells in the spleen was determined by FACS analysis at 36 hours. Each triangle represents an individual mouse and the bar indicates the mean. *P-values ≤ 0.05 were generated by Wilcoxon Rank-Sum test. **(D)** B6 mice (n = 4/group) were injected once i.p. with G-CSF/anti-G-CSF mAb complexes (1.5 μg G-CSF plus 7.5 μg anti-G-CSF mAb), cytokine alone (1.5 μg G-CSF), pegylated G-CSF (3.1 μg PEG-G-CSF), or vehicle alone. Peripheral blood was harvested prior to injection and on days 1, 2, 4, and 6. The percentage of CD11b^+^Gr-1^+^ myeloid cells was determined by FACS analysis. The error bars indicate standard deviation. *P-values < 0.01 were generated by a repeated measures design with group-time interactions tested at each timepoint.

To test the efficacy of G-CSF/anti-G-CSF mAb complexes, B6 mice were injected i.p. once with complexes containing 0.5 μg G-CSF pre-associated with 2.5 μg anti-G-CSF mAb (clone BVD11-37G10). We observed that G-CSF/anti-G-CSF mAb complexes induced a marked increase in the percent of splenic CD11b^+^Gr-1^+^ myeloid cells (Figure 1A). This effect was more dramatic after three injections of G-CSF/anti-G-CSF mAb complexes (1.5 μg G-CSF and 7.5 μg anti-G-CSF mAb) over 1 week, which induced over a 20-fold increase in the number of CD11b^+^Gr-1^+^ myeloid cells per spleen (Figure 1B). Importantly, administration of cytokine or antibody alone did not significantly alter the number of CD11b^+^Gr-1^+^ myeloid cells. In addition to spleen, we observed increased frequencies of CD11b^+^Gr-1^+^ myeloid cells in the blood, bone marrow, lung, but not the lymph node (Additional file 1: Figure S1). The phenotype of these expanded myeloid cells is consistent with that of neutrophils (or neutrophilic granulocytes), a cell population known to be G-CSF responsive [1,40,41]. As previously observed, these myeloid cells exhibited a higher FSC/SSC profile indicating a larger and more granular cell morphology compared with other lymphocytes [42]. Furthermore, of the two commonly described CD11b^+^Gr-1^+^ subpopulations [43,44], the G-CSF/anti-G-CSF mAb complex-expanded population was Ly6G^+^Ly6C^low^ and not Ly6G^-^Ly6C^hi^ (Additional file 1: Figure S2). Titration of G-CSF/anti-G-CSF mAb complexes versus G-CSF alone revealed that 0.015 μg of G-CSF complexed with anti-G-CSF mAb was more biologically active than 1.5 μg of G-CSF alone, indicating a ~100-fold increase in biological activity upon association of G-CSF with anti-G-CSF mAb (Figure 1C). Furthermore, enhanced frequencies of myeloid cells persisted for 4 days after administration of the cytokine complex before declining (Figure 1D). In contrast, the administration of an equal molar dose of either free G-CSF or pegylated G-CSF led to markedly lower responses, with elevated myeloid cells persisting for only one day with free G-CSF and for 2 days with pegylated G-CSF. We also observed that pre-association of anti-G-CSF mAb could directly enhance the biological activity of pegylated G-CSF as measured by increased numbers of myeloid cells in the spleen following a single injection of pegylated cytokine and/or antibody (Additional file 1: Figure S3).

While G-CSF/anti-G-CSF mAb complexes induced the potent expansion of CD11b^+^Gr-1^+^ myeloid cells, it was important to assess whether other cell populations were affected. To maximize any changes observed upon administration of cytokine complexes, we treated mice with G-CSF/anti-G-CSF mAb complexes every 48 hours for 20 days. We observed no significant differences in the frequencies or numbers of B cells (B220^+^), T cells (CD8^+^ or CD4^+^), or dendritic cells (CD11c^+^) among lymphocyte-gated cells (Additional file 1: Figure S4). Again, CD11b^+^Gr-1^+^ myeloid cells were expanded systemically in spleen, peripheral blood, and bone marrow (Additional file 1: Figure S4B & C). Also, as expected based on studies with G-CSF alone [8,9], in mice treated every 48 hours for 1 week with G-CSF/anti-G-CSF mAb complexes, we observed increases in hematopoietic progenitor cells in the spleen and peripheral blood identified as lineage negative and expressing Sca1 and c-Kit (Additional file 1: Figure S5). It is also relevant that we did not observe significant changes in hematocrit levels (Additional file 1: Figure S6), suggesting a lack of toxicity consistent with previous observations using G-CSF transgenic mice [45].

To verify the specificity of G-CSF/anti-G-CSF mAb complexes for CD11b^+^Gr-1^+^ myeloid cells and to assess whether these complexes induced proliferation of other cell populations, we treated mice with BrdU during administration of cytokine complexes or controls. We observed an enhanced percentage of BrdU^+^ CD11b^+^Gr-1^+^ myeloid cells but not other cell populations in mice treated with G-CSF/anti-G-CSF mAb complexes (Additional file 1: Figure S7). G-CSF administration alone had no effect. As an additional control, injection of mice with IL-15/sIL-15Rα-Fc cytokine complexes [35-37] induced proliferation of CD8^+^ T cells but not myeloid cells. Cumulatively, these results suggest that G-CSF/anti-GCSF mAb complexes act directly to specifically induce proliferation of CD11b^+^Gr-1^+^ myeloid cells or their precursors. However, we cannot formally exclude the possibility that G-CSF/anti-GCSF mAb complexes induce the localization of dividing cells into the periphery [46].

**Figure 2:**
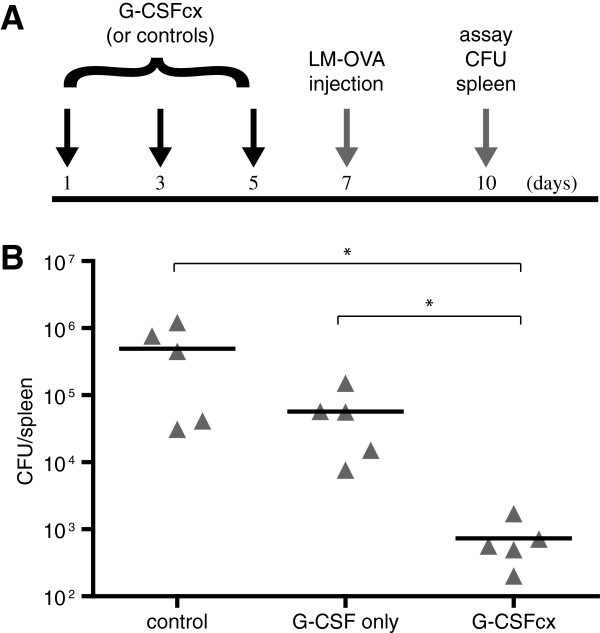
**G-CSF/anti-G-CSF mAb complexes enhance immunity against bacterial infection. (A)** B6 mice (n = 5/group) were treated i.p. on days 1, 3, and 5 with G-CSF/anti-G-CSF mAb complexes (1.5 μg G-CSF plus 7.5 μg anti-G-CSF mAb), G-CSF alone (1.5 μg G-CSF), or vehicle alone. On day 7, mice were injected i.v. with 1x10^5^ colony-forming units (CFU) of Listeria monocytogenes (LM-OVA). **(B)** On day 10, spleens were harvested and the number of CFU was determined. The triangles indicate individual mice and the bar indicates the average per group. *P-values ≤ 0.05 were generated by Wilcoxon Rank-Sum test.

To determine if the phenotypic changes induced by administration of G-CSF/anti-G-CSF mAb complexes were functionally relevant, we assessed whether administration of cytokine complexes would lead to heightened protective immunity against Listeria monocytogenes. In contrast to mice injected with free G-CSF alone (a subtherapeutic dose) or vehicle alone, mice injected with G-CSF/anti-G-CSF mAb complexes demonstrated a roughly 100-fold reduction in bacterial counts within the spleen at day 3 post-infection (Figure 2).

### No evidence of G-CSF-mediated suppression of CD8^+^ T cell immune responses

**Figure 3:**
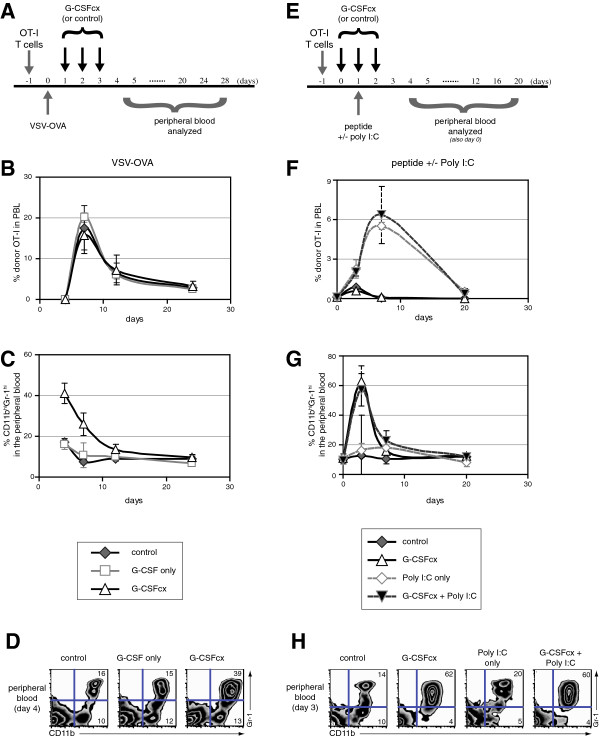
**CD8**^**+ **^**T cell responses are normal in the context of G-CSF/anti-G-CSF mAb-mediated expansion of CD11b**^**+**^**Gr-1**^**+ **^**myeloid cells. (A)** OT-I CD8^+^ T cells (10^5^) were transferred to B6 mice and infected with VSV-OVA one day later. On day 1, day 2, and day 3 post-infection, mice were treated i.p. with G-CSF/anti-G-CSF mAb complexes (1 μg G-CSF plus 5 μg anti-G-CSF mAb), G-CSF alone (1 μg G-CSF), or vehicle alone (n = 4/group). **(B)** Peripheral blood was harvested at the timepoints indicated and the percentage of donor OT-I CD8^+^ T cells among lymphocytes was assessed. **(C)** As in 'B’ except the percentage of myeloid cells (CD11b^+^Gr-1^+^) among peripheral blood cells was assessed. **(D)** Representative data from 'C’ on day 4. **(E)** OT-I CD8^+^ T cells (10^6^) were transferred to B6 mice on day -1. On day 0, day 1, and day 2, mice were injected i.p. with G-CSF/anti-G-CSF complexes (1.5 μg G-CSF plus 7.5 μg anti-G-CSF mAb). On day 1, mice also received 50 μg of OVA peptide i.v. followed shortly afterwards with one injection i.p. of poly I:C (n = 2-3/group). **(F)** Peripheral blood was harvested at the time-points indicated and the percentage of donor OT-I CD8^+^ T cells among lymphocytes was assessed. **(G)** As in 'F’ except the percentage of myeloid cells (CD11b^+^Gr-1^+^) among peripheral blood cells was assessed. **(H)** Representative data from 'G’ on day 3. The error bars in 'B’, 'C’, 'F’, and 'G’, indicate standard deviation.

It is becoming increasingly well recognized that effective cancer therapy often requires the expansion and survival of CD8^+^ tumor-specific T cell response [47]. While expansion of myeloid cells during cytoreductive therapy in cancer patients is important for immunity to infection, in some settings, certain myeloid cell subpopulations have immunosuppressive activity [48,49]. Thus, an important question is whether the dramatic increase in CD11b^+^Gr-1^+^ myeloid cells or other biological changes following administration of a high dose of G-CSF might suppress CD8^+^ T cell responses [50,51]. To assess this possibility, we evaluated the response of antigen-specific OT-I donor T cells following infection with vesicular stomatitis virus genetically engineered to express ovalbumin (VSV-OVA). In this model, the OVA-specific donor CD8^+^ T cells undergo an expansion, a contraction, and a memory phase following infection (Figure 3A) [39]. We found that administration of G-CSF/anti-G-CSF mAb complexes during the first three days of infection did not alter the kinetics of the CD8^+^ T cell response (Figure 3B), despite a nearly 3-fold increase in the percentage of CD11b^+^Gr-1^+^ myeloid cells in the peripheral blood (Figure 3C, D). As VSV-OVA represents an extremely potent method for activating naïve OT-I T cells, we also assessed the impact of CD11b^+^Gr-1^+^ myeloid cell expansion on antigen-specific OT-I CD8^+^ T cell responses after stimulation with OVAp and poly I:C, a less effective method of inducing T cell expansion (Figure 3E). Similarly, administration of G-CSF/anti-G-CSF mAb complexes did not affect the expansion of OT-I CD8^+^ T cells induced by OVAp and poly I:C vaccination despite the dramatic accumulation of CD11b^+^Gr-1^+^ myeloid cells (Figure 3F, G, & H). Finally, using an in vitro assay, splenocytes from G-CSF/anti-G-CSF mAb complex-treated mice did not suppress IL-2-induce proliferation of activated CD8^+^ T cells (Additional file 1: Figure S8).

### G-CSF/anti-G-CSF mAb complexes enhance CD11b^+^Gr-1^+^ myeloid cell recovery after cytoreductive therapy

**Figure 4:**
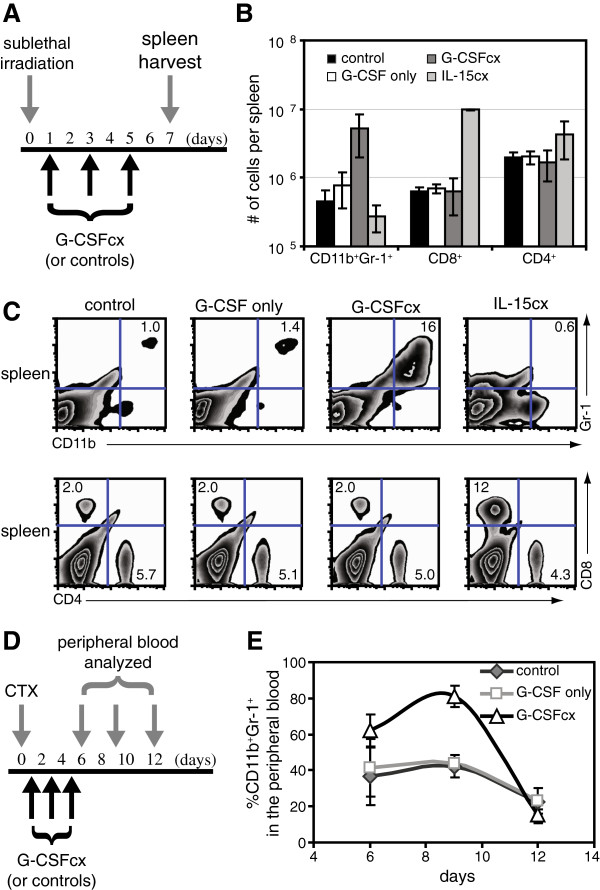
**G-CSF/anti-G-CSF mAb complexes expand CD11b**^**+**^**Gr-1**^**+ **^**myeloid cells after cytoreductive therapy. (A)** B6 mice were sublethally irradiated (600 rad) on day 0 and treated i.p. on day 1, day 3, and day 5 with G-CSF/anti-G-CSF mAb complexes (1 μg G-CSF plus 5 μg anti-G-CSF mAb), G-CSF alone (1 μg G-CSF), vehicle alone, or IL-15/sIL-15Rα complexes (1.5 μg mIL-15 plus 7 μg sIL-15Rα-Fc) (n = 2/group). **(B)** Spleen cells were harvested on day 7 and analyzed by FACS analysis, and the absolute number of cells was determined. **(C)** Flow cytometric data from 'B’. CD11b and Gr-1 staining (top row) was gated with a large FSS/SSC gate, while CD8 and CD4 staining (bottom row) was gated with a tight lymphocyte gate. **(D)** B6 mice were treated with cyclophosphamide (4 mg) on day 0 and treated i.p. on day 1, day 3, and day 5 with G-CSF/anti-G-CSF mAb complexes (1 μg G-CSF plus 5 μg anti-G-CSF mAb), G-CSF alone (1 μg G-CSF), or vehicle alone (n = 4/group). **(E)** The percentage of CD11b^+^Gr-1^+^ within the peripheral blood. All data are representative of 2 independent experiments.

As the mechanism of action for antibody cytokine complexes is not precisely known, it is possible that cytoreductive therapies might ablate host cells mechanistically important in the improved biological activity exhibited by cytokine complexes. We tested this possibility in mice after sublethal irradiation or cyclophosphamide administration. In both cases, administration of G-CSF/anti-G-CSF mAb cytokine complexes, but not G-CSF alone, induced the potent expansion of CD11b^+^Gr-1^+^ myeloid cells (Figure 4A-E). As an additional control, administration of IL-15/sIL-15Rα-Fc complexes after sublethal irradiation induced the expansion of CD8^+^ T cells but not CD11b^+^Gr-1^+^ myeloid cells. Cumulatively, these results demonstrate that cytoreductive therapy does not result in the destruction of cells essential to the enhanced activity of G-CSF/anti-G-CSF mAb complexes.

In addition to promoting myeloid recovery during standard cytoreductive therapy, G-CSF also facilitates myeloid recovery after bone marrow transplantation in human patients [52]. To assess whether G-CSF/anti-GCSF mAb complexes could also facilitate the recovery of myeloid cells during bone marrow transplantation, we reconstituted lethally irradiated CD45.1 mice with B6 bone marrow. Mice were then treated every 48 hours with G-CSF/anti-GCSF mAb complexes, G-CSF alone, or vehicle alone, for one or three weeks. We observed striking CD11b^+^Gr-1^+^ myeloid cell recovery at both the one and three week time-points (Additional file 1: Figure S9A & B). These expanded myeloid cells represented both host and donor cells (Additional file 1: Figure S9B), indicating that G-CSF/anti-G-CSF mAb complexes exhibited broad activity. Notably, at three weeks following bone marrow transplantation, spleens were significantly enlarged in mice treated with G-CSF/anti-G-CSF mAb complexes versus G-CSF alone or vehicle alone (Additional file 1: Figure S9C). This increase in spleen size was largely attributable to increased numbers of CD11b^+^Gr-1^+^ myeloid cells (Additional file 1: Figure S9D).

While G-CSF/anti-G-CSF mAb complexes enhanced myeloid cell recovery, these complexes failed to facilitate the recovery of the lymphoid cells such as CD8^+^ T cells (Figure 4, Additional file 1: Figures S4, & S7). Thus, we asked whether we might see a more complete hematopoeitic recovery during bone marrow transplantation by combining G-CSF/anti-GCSF mAb complexes with an agent capable of driving CD8^+^ T cell recovery, such as IL-15/sIL-15Rα-Fc complexes. Consistent with this hypothesis, after bone marrow transplantation, we observed the recovery of both myeloid and lymphoid cell populations only after treatment with both G-CSF/anti-G-CSF mAb complexes and IL-15/sIL-15Rα-Fc complexes (Additional file 1: Figure S10).

### Enhanced recovery of CD11b^+^Gr-1^+^ myeloid cells following administration of G-CSF/anti-G-CSF mAb complexes does not impair antigen-specific CD8^+^ T cell responses during myelosuppression

**Figure 5:**
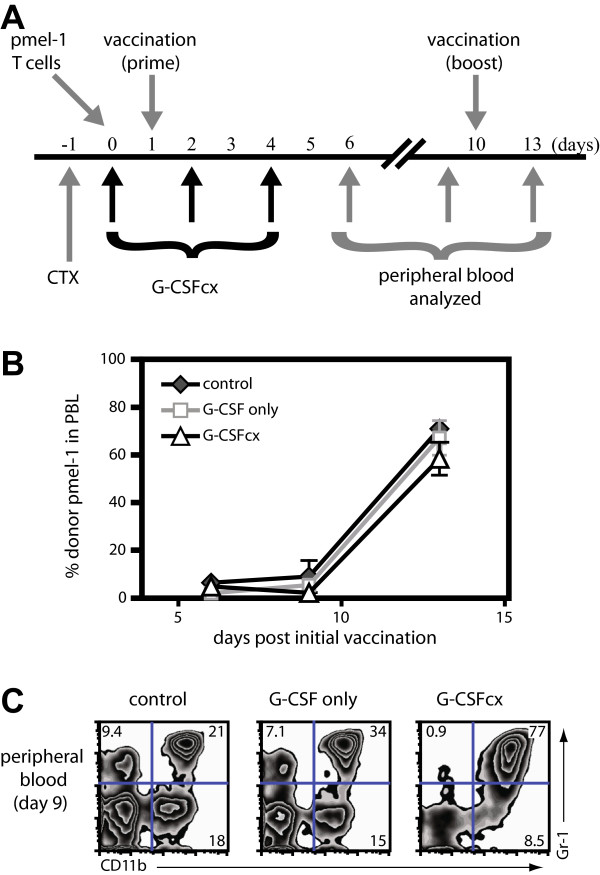
**G-CSF/anti-G-CSF mAb-mediated expansion of CD11b**^**+**^**Gr-1**^**+ **^**myeloid cells during myelosuppression does not impair antigen-specific CD8**^**+ **^**T cell proliferative responses. (A)** B6 mice (n = 4/group) were treated with 4 mg cyclophosphamide (CTX) on day -1 and adoptively transferred with 10^6^ pmel-1 CD8^+^ T cells on day 0. On days 1 and 10, mice received a prime boost vaccination s.c. with gp100_25-33_ peptide (100 μg, i.v.) concomitant with poly I:C (200 μg, i.p.). On day 0, day 2, and day 4, mice were treated i.p. with G-CSF/anti-G-CSF mAb complexes (1 μg G-CSF plus 5 μg anti-G-CSF mAb), G-CSF alone (1 μg G-CSF), or vehicle alone (n = 4/group). **(B)** Peripheral blood was harvested at the timepoints indicated and the percentage of donor pmel-1 CD8^+^ T cells among lymphocytes was assessed by flow cytometry. **(C)** The percentage of myeloid cells (CD11b^+^Gr-1^+^) among peripheral blood was assessed at day 9. All data are representative of 2 independent experiments.

Although we established that elevated frequencies of CD11b^+^Gr-1^+^ myeloid cells did not inhibit CD8^+^ T cell responses in normal mice, it was important to assess these parameters during cytopenic conditions which could theoretically favor the G-CSF-mediated development of suppressive myeloid cells. Thus, we utilized an antigen-specific vaccination model that we have shown induces effective anti-tumor CD8^+^ T cell immunity in the context of cyclophosphamide (CTX)-induced immunosuppresions [53]. Mice were treated with CTX, adoptively transferred with naïve pmel-1 CD8^+^ T cells, and then given a prime/boost vaccination with the gp100_25-33_ peptide/poly I:C as outlined in Figure 5A. Mice were treated with G-CSF/anti-G-CSF mAb complexes for 5 days after CTX treatment and during the priming phase. As shown in Figure 5B, independent of administration of G-CSF/anti-G-CSF mAb complexes, the prime/boost vaccination induced a vigorous expansion of pmel-1 CD8^+^ T cells. The effectiveness of the G-CSF/anti-G-CSF mAb complexes was apparent from the potent expansion of CD11b^+^Gr-1^+^ myeloid cells observed in the peripheral blood at day 9 following CTX treatment (Figure 5C). These results demonstrate that even in the context of cytoreductive therapy as may occur during cancer therapy, the increased numbers and percentages of CD11b^+^Gr-1^+^ myeloid cells in the periphery did not inhibit the expansion of antigen-specific CD8^+^ T cells.

## Discussion

While the administration of G-CSF-related drugs provides a highly effective strategy for maintaining innate immunity in the context of cytoreductive cancer therapies, these drugs are expensive and exhibit limited biological activity. In this study, we find that the biological activity of G-CSF can be dramatically enhanced, even more so than with pegylation, by pre-association with an anti-G-CSF mAb (Figure 1). Administration of G-CSF/anti-G-CSF mAb complexes specifically drove the expansion of CD11b^+^Gr-1^+^ myeloid cells without affecting other major lineages. The phenotype of these CD11b^+^Gr-1^+^ cells is consistent with neutrophils, a cell population known to be G-CSF responsive [1,40,41] and whose depletion during certain cancer therapies is thought to contribute to the development of opportunistic infection. Due to the limitations of currently available G-CSF compounds, we evaluated the preclinical potential of G-CSF/anti-G-CSF mAb complexes during myelosuppresion. We found that G-CSF/anti-G-CSF mAb complexes facilitated the rapid recovery of CD11b^+^Gr-1^+^ myeloid cells during sublethal irradiation, cyclophosphamide treatment, or bone marrow transplantation (Figure 4 & Additional file 1: Figure S9). Furthermore, pretreatment with G-CSF/anti-G-CSF mAb complexes bestowed significantly greater protection against bacterial infection than pretreatment with G-CSF alone (Figure 2). Finally, effective CD8^+^ T cell immune responses were not compromised following administration of G-CSF/anti-G-CSF mAb complexes, even during cyclophosphamide-induced myelosuppression (Figure 3 &5).

Perhaps the most important finding in our study was the ability of G-CSF/anti-G-CSF mAb complexes to act more potently *in vivo* than pegylated G-CSF (Figure 1D). Our study represents the first comparison of antibody complex technology with pegylation. While pegylation is used to increase the activity of a number of FDA-approved protein therapeutics, antibody cytokine technology has not yet been translated to clinical use. Our findings suggest that antibody cytokine complex technology will be useful for improving the biological activity for a range of protein therapeutics. In this regard it is relevant to note that antibody cytokine complexes might also be a particularly useful technology for patients with anti-PEG antibodies [25-29].

Based on current indications for G-CSF-related drugs, we envision multiple clinical applications for G-CSF/anti-G-CSF mAb complexes. Foremost, these cytokine complexes might provide an improved method for facilitating myeloid cell recovery during cancer therapy or other myelosuppressive disorders, and thus, reduce the incidence of opportunistic infections. Administration of G-CSF/anti-G-CSF mAb complexes may also allow the administration of higher doses of neutropenia-inducing anti-tumor therapies. Another application of G-CSF/anti-G-CSF complexes could be in the improved mobilization of progenitor cells in the peripheral blood. This latter possibility needs further investigation as prolonged and increased biological activity of G-CSF may not be desirable in healthy individuals or could alter the expected distribution of progenitor cells in the blood.

In addition to enhancing the efficacy of G-CSF for existing conditions, the increased potency of G-CSF/anti-G-CSF mAb complexes versus existing G-CSF-based drugs may offer other advantages. For example, the maximal potential expansion of CD11b^+^Gr-1^+^ myeloid cells might be greater with G-CSF/anti-G-CSF mAb complexes than with currently available G-CSF-based drugs. This may suggest alternate applications for G-CSF/anti-G-CSF mAb complexes not necessarily evident from previous studies with G-CSF. For example, burn patients or others at high risk for infection might benefit by administration of G-CSF/anti-G-CSF mAb complexes. Consistent with this possibility, we found enhanced protection against bacterial infection with antibody cytokine complexes (Figure 2) and elevated frequencies of myeloid cells 1–3 days longer than with G-CSF alone or PEG-G-CSF alone (Figure 1D).

The precise mechanism of how G-CSF/anti-G-CSF mAb complexes and other cytokine complexes mediate improved activity versus free cytokine is not known. However, in the case of many cytokines complexes, an increase in cytokine half-life *in vivo* has been observed [32]. Thus, it is likely that binding of anti-G-CSF mAb to G-CSF improves the half-life of G-CSF, and thus, accounts for part of the enhanced biological activity. However, other mechanisms could be relevant including the ability of antibody binding to protect the cytokine from degradation or to impact cytokine localization. It is notable that free G-CSF appeared to yield relatively improved responses in the bone marrow versus other locations. This could be a consequence of the localization of Fc-expressing cells or that G-CSF-matured neutrophils are migrating out of the bone marrow. These and other possibilities represent important areas for future research.

It is worth noting that while G-CSF/anti-G-CSF mAb complexes may prove useful clinically, this technology may act synergistically with other therapeutic strategies. Thus, for example, as pegylation likely acts in distinct mechanisms, our results suggest that combining pegylated G-CSF with anti-G-CSF mAb could lead to synergistic improvements in biological activity. Our findings also suggest that combining administration of G-CSF with agents such as IL-15 that promote lymphoid cell expansion might also be advantageous. All of these possibilities may allow for the administration of more effective regimens of cytotoxic anti-cancer therapies.

## Conclusion

Our results demonstrate a novel and improved method for increasing the biological activity of G-CSF. In addition to showing superior activity than pegylated G-CSF, G-CSF/anti-G-CSF mAb complexes may be advantageous for patients with anti-PEG antibodies. Our results also suggest that the use of antibody cytokine complexes methodology may be useful for increasing the biological activity of other cytokines, such as GM-CSF, currently used to facilitate hematopoietic recovery.

## Methods

### Generation of cytokine complexes

Human (h) G-CSF (Neupogen) and PEG-G-CSF (Neulasta) were purchased from the UCSD pharmacy (La Jolla, CA). Anti-hG-CSF mAb (clone BVD11-37G10) was purchased from SouthernBiotech (Birmingham, AL). Cytokine complexes were generated by incubation of the cytokine and antibody for 20 minutes at 37°C and diluted at least 10-fold in phosphate-buffered saline before injection (i.p. or i.v.). G-CSF/anti-G-CSF mAb complexes were generated at a 1:5 ratio of cytokine to antibody (1 μg of cytokine pre-associated with 5 μg of antibody), a ratio that ensures all cytokine is pre-bound with antibody.

### Mice and *in vivo* protocols

C57BL/6 (B6), B6.Ly5.1 (CD45.1), RAG1^-/-^, OT-I, and pmel-1 mice were purchased from the Jackson Laboratory (Bar Harbor, ME). All animal work was performed under institutional and federal guidelines outlined by the University of California, San Diego and the Medical University of South Carolina. Bacterial protection and the determination of colony forming units were performed using Listeria monocytogenes expressing ovalbumin (LM-OVA) as previously described [54]. For experiments using cells from OT-I TCR transgenic mice, 10^5^ or 10^6^ naive OT-I CD8^+^ T cells were injected i.v. one day before antigenic challenge. For infections, mice were injected i.v. with 10^5^ plaque forming units of vesicular stomatitis virus expressing ovalbumin (VSV-OVA) [55]. For peptide immunization, mice were injected i.v. on day 0 with 50 μg of SIINFEKL (OVAp) peptide (American Peptide Company, Sunnyvale, CA). Where indicated, on days 0 and 1 mice received injections i.p. with 100 μg of Poly I:C (InvivoGen, San Diego, CA). For experiments using cells from pmel-1 TCR transgenic mice, immunizations were performed as we have previously described [53]. Briefly, 10^6^ naïve pmel-1 CD8^+^ T cells were injected i.v. one day after treatment with 4 mg of cyclophosphamide (Sigma, St. Louis, MO). One and ten days after pmel-1 transfer, mice were immunized s.c. with 100 μg of EGSRNQDWL (gp100_25-33_) peptide (American Peptide) and 200 μg of poly I:C. For bone marrow transplantation experiments, mice were lethally irradiated (1400 rad) and adoptively transferred with 2x10^6^ T- and B-depleted CD45.1 congenic bone marrow cells as previously described [56]. For measurement of proliferation with Bromodeoxyuridine (BrdU; Sigma), mice were injected i.v. with BrdU (2 mg) and maintained on BrdU drinking water (0.8 mg/ml) as previously described [57].

### Flow cytometric analysis

Cells were analyzed by flow cytometry using standard techniques. Briefly, cells were washed in staining buffer (PBS, 2% bovine growth serum and 0.01% sodium azide) and stained with fluorescently labeled antibodies as indicated. The antibodies used in these studies were as follows: B220-FITC and- PerCP (RA3-6B2; eBioscience, San Diego, CA), CD3-PE-Cy5 (145-2C11, eBioscience); CD4-FITC (RM4-5, eBioscience), CD8-FITC and –PerCP (53–6.7; BD Biosciences PharMingen, San Diego, CA), CD11b-PE and –Pe-Cy5 (M1/70; eBioscience), CD11c-PE and –APC (N418, eBioscience),CD25-FITC and phycoerythrin (PE; PC61.5; eBioscience), CD44-allophycocyanin (APC; IM7; eBioscience), CD122-PE (TM-β1, eBioscience), CD45.1-FITC, -PE, and –APC (A20, eBioscience), CD45.2-FITC and –APC (104; eBioscience),CD49b-PE and -APC (DX5; eBioscience); c-kit-PE (2B8, BD Biosciences), Gr-1-FITC and PE-Cy5 (RB6-85C; eBioscience), Ly6C- PE (HK1.4, eBioscience), Ly6G-biotin (1A8, Biolegend), NK1.1-PE (PK136; eBioscience), Sca-1-FITC (D7, eBioscience), and TER-119-PE-Cy5.5 (TER-119; eBioscience). Staining for intracellular bromodeoxyuridine (BrdU) was performed with reagents and according to the instructions outlined in the FITC BrdU flow kit (559519, BD Biosciences). Flow cytometry was performed with a BD FACS Caliber and BD Accuri (BD Biosciences). Data were analyzed using FlowJo software (TreeStar, San Carlos, CA).

### Statistical methods

The Wilcoxon Rank-Sum test (the non-parametric alternative to the two-sample t-test for non-normal data) was used to determine the statistically significant difference between the underlying distributions of two groups. For measurements taken over time we fitted a linear mixed model with a log-transformed outcome and the following effects: study group (treatment), time, and group-time interaction. Three variance-covariance structures were tested. The covariance that resulted in the smallest Akaike’s information criterion was used. In all cases it was the compound symmetry structure. If the overall group-time interaction showed to be significant, we further explored the differences in the levels of that interaction. We performed comparisons of the group levels within each timepoint. All the analyses were carried out using SAS 9.2 and R 3.0.1 software. Throughout the paper all P-values presented are two-sided with 0.05 or less considered significant.

## Abbreviations

BrdU: Bromodeoxyuridine; CTX: Cyclophosphamide; LM-OVA: Listeria monocytogenes expressing ovalbumin; mAb: Monoclonal antibody; PEG: Polyethylene glycol; sIL-15Rα: Soluble IL-15 receptor α; OVAp: SIINFEKL peptide; VSV-OVA: Vesicular stomatitis virus genetically engineered to express ovalbumin.

## Competing interests

Authors (MPR, MLS, DJC, AWG) are inventors on a provisional patent application associated with the application of G-CSF/anti-G-CSF mAb complexes.

## Authors’ contributions

MPR and MLS conceived the study, participated in the design, conducted experiments, wrote and edited the manuscript. DJC and AWG conceived the study, participated in the design, wrote and edited the manuscript. AD, CJM, and GLR participated in the design, conducted experiments, and edited the manuscript. CC performed statistical analysis and edited the manuscript. All authors read and approved the final manuscript.

## Supplementary Material

Additional file 1: Figure S1 G-CSF/anti-G-CSF mAb complexes induce the expansion of CD11b^+^Gr-1^+^ myeloid cells. **Figure S2.** Administration of G-CSF/anti-G-CSF mAb complexes induces CD11b^+^Gr-1^+^ myeloid cells with a Ly6G^+^Ly6C^low^ phenotype. **Figure S3.** Pre-association of anti-G-CSF mAb with pegylated G-CSF improves biological activity. **Figure S4.** Long-term administration of G-CSF/anti-G-CSF mAb complexes induces splenomegaly and dramatic expansion of CD11b^+^Gr-1^+^ myeloid cells. **Figure S5.** Administration of G-CSF/anti-G-CSF mAb complexes induces increased numbers of hematopoietic progenitor cells in the spleen and peripheral blood. **Figure S6.** Extended administration of G-CSF/anti-G-CSF mAb complexes does not affect hematocrit. **Figure S7.** G-CSF/anti-G-CSF mAb complexes induce the proliferation of CD11b^+^Gr-1^+^ myeloid cells. **Figure S8.** Activated CD8^+^ T cells proliferate normally when mixed with splenocytes from G-CSF/anti-G-CSF mAb complex-treated mice. **Figure S9.** G-CSF/anti-G-CSF mAb complexes expand CD11b^+^Gr-1^+^ myeloid cells after bone marrow transplantation. **Figure S10.** The combination of G-CSF/anti-G-CSF mAb complexes and IL-15/sIL-15Rα-Fc complexes induces more effective hematopoietic recovery following bone marrow transplantation. Click here for file
