# Supplementary material for: G-CSF/anti-G-CSF antibody complexes drive the potent recovery and expansion of CD11b+Gr-1+ myeloid cells without compromising CD8+ T cell immune responses
Source: J Hematol Oncol. 2013 Oct 1;6:75. doi: 10.1186/1756-8722-6-75 (PMC3850648; doi:10.1186/1756-8722-6-75)
Supplement: Additional file 1: Figure S1 — G-CSF/anti-G-CSF mAb complexes induce the expansion of CD11b+Gr-1+ myeloid cells. Figure S2. Administration of G-CSF/anti-G-CSF mAb complexes induces CD11b+Gr-1+ myeloid cells with a Ly6G+Ly6Clow phenotype. Figure S3. Pre-association of anti-G-CSF mAb with pegylated G-CSF improves biological activity. Figure S4. Long-term administration of G-CSF/anti-G-CSF mAb complexes induces splenomegaly and dramatic expansion of CD11b+Gr-1+ myeloid cells. Figure S5. Administration of G-CSF/anti-G-CSF mAb complexes induces increased numbers of hematopoietic progenitor cells in the spleen and peripheral blood. Figure S6. Extended administration of G-CSF/anti-G-CSF mAb complexes does not affect hematocrit. Figure S7. G-CSF/anti-G-CSF mAb complexes induce the proliferation of CD11b+Gr-1+ myeloid cells. Figure S8. Activated CD8+ T cells proliferate normally when mixed with splenocytes from G-CSF/anti-G-CSF mAb complex-treated mice. Figure S9. G-CSF/anti-G-CSF mAb complexes expand CD11b+Gr-1+ myeloid cells after bone marrow transplantation. Figure S10. The combination of G-CSF/anti-G-CSF mAb complexes and IL-15/sIL-15Rα-Fc complexes induces more effective hematopoietic recovery following bone marrow transplantation. [file 1756-8722-6-75-S1.doc]

**
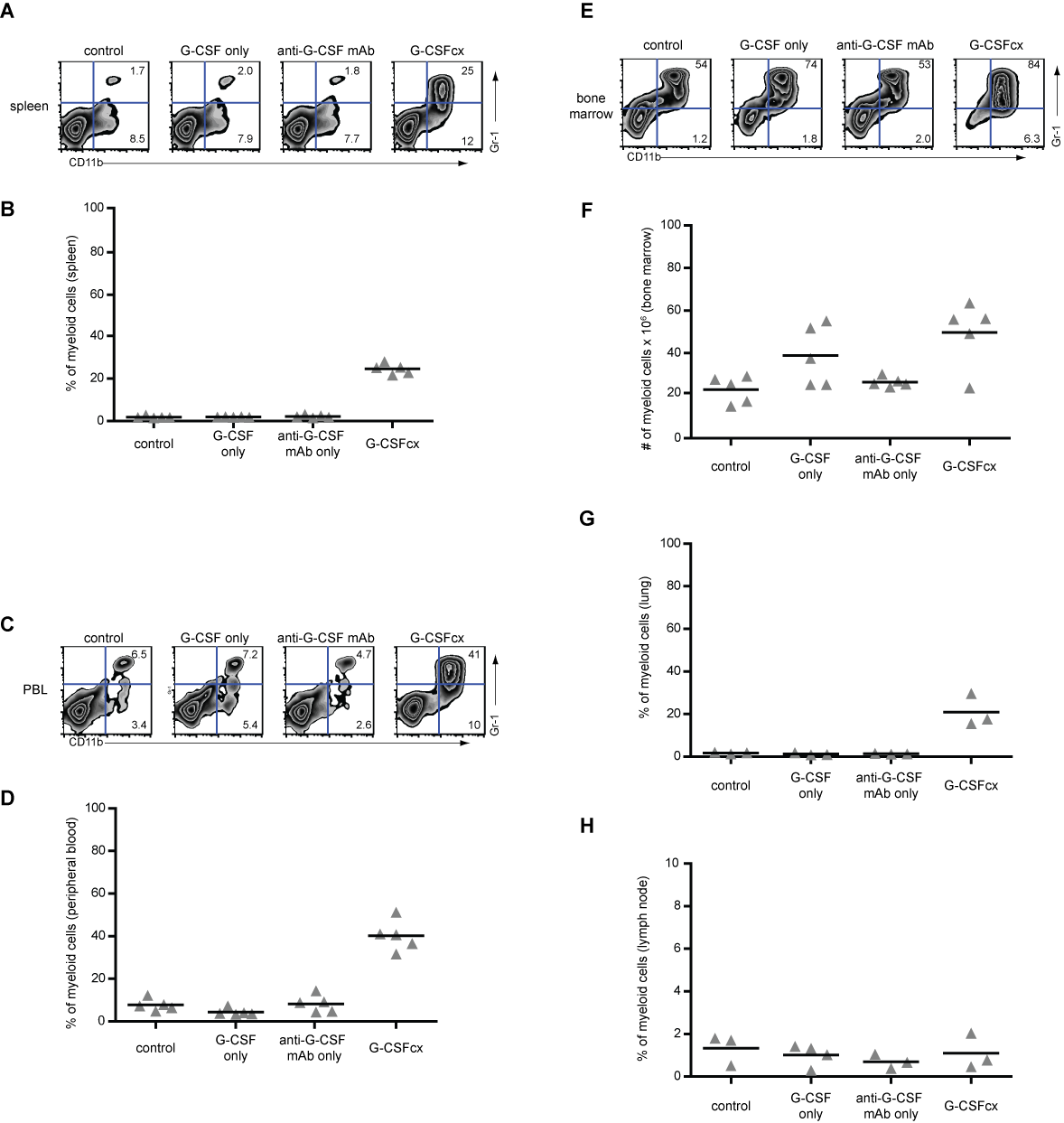
**

Figure S1. G-CSF/anti-G-CSF mAb complexes induce the expansion of CD11b^+^GR1^+^ myeloid cells. B6 mice were treated as described for Figure S1B. The percentage of myeloid cells is shown for the spleen (A&B), peripheral blood (C&D), bone marrow (E), lung (G), and lymph node (H). The number of bone marrow cells is indicated in (F). For (B, D, F, G, & H), each triangle represents an individual mouse and the bar indicates the mean.

(Figure S1 continued)


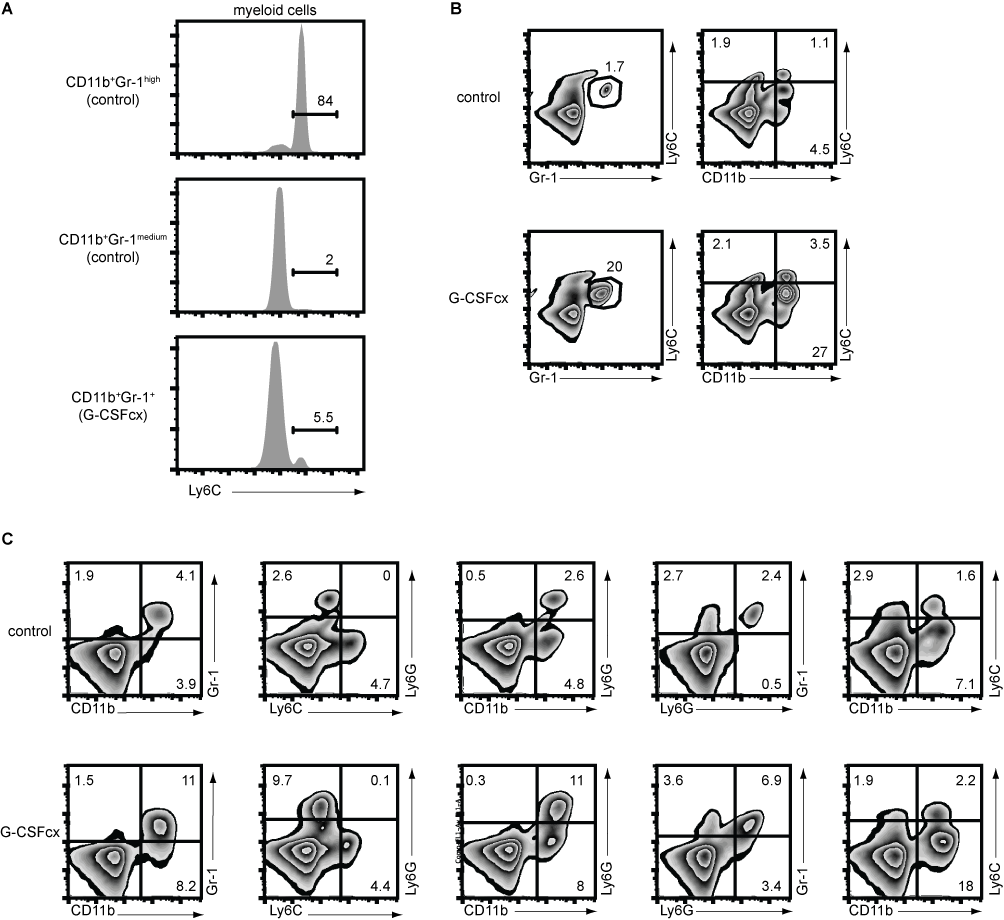


Figure S2. Administration of G-CSF/anti-G-CSF mAb complexes induces CD11b^+^Gr-1^+^ myeloid cells with a Ly6G^+^Ly6C^low^ phenotype. (A) B6 mice were injected on days 1, 2, and 3 with G-CSF/anti-G-CSF mAb complexes (1 µg G-CSF plus 5µg anti-G-CSF mAb) or vehicle alone. On day 4, spleens were harvested and analyzed by flow cytometry for expression of Gr-1, CD11b, and Ly6C (clone HK1.4). Histograms are gated on the cell population indicated to the left. (B) Shows same conditions as in ‘A’ except gated on total cells within a large FSC/SSC gate. (C) Flow cytometry results from a second experiment identical to ‘A’ except cells were stained for Gr-1, CD11b, Ly6C (clone HK1.4), and Ly6G (clone 1A8). Plots are gated on total cells within a large FSC/SSC gate. All results are representative of at least 2 mice per condition.


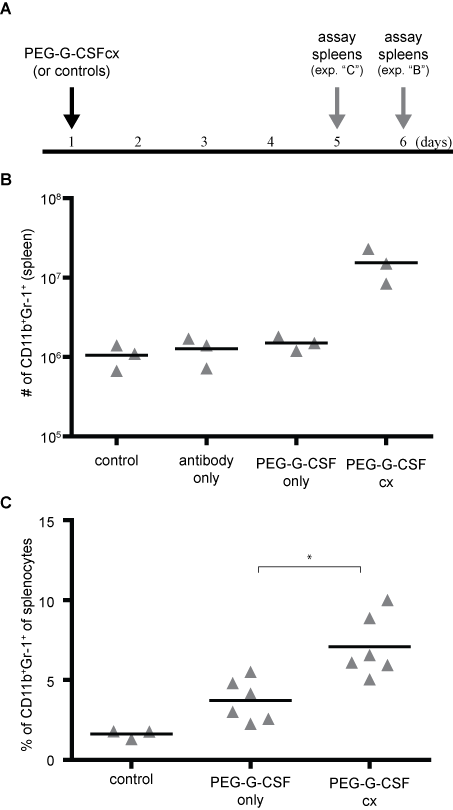


Figure S3. Pre-association of anti-G-CSF mAb with pegylated G-CSF improves biological activity. (A) Shows the timeline for the experiments shown in ‘B’ and ‘C’. (B) B6 mice were treated i.p. with PEG-G-CSF/anti-G-CSF complexes (4µg PEG-G-CSF plus 10µg anti-G-CSF mAb), PEG-G-CSF only (4µg PEG-G-CSF), antibody alone (10µg anti-G-CSF mAb), or vehicle alone. Five days later, spleens were harvested, and the absolute number of myeloid cells (CD11b^+^Gr-1^+^) was determined. The triangles indicate individual mice and the bar indicates the average per group. (C) B6 mice were treated i.p. with PEG-G-CSF/anti-G-CSF complexes (2µg PEG-G-CSF plus 5µg anti-G-CSF mAb), PEG-G-CSF only (2µg PEG-G-CSF), or vehicle alone. Four days later, spleens were harvested, and the percentage of myeloid cells (CD11b^+^Gr-1^+^) was determined. The triangles indicate individual mice and the bar indicates the average per group. *P-values ≤ 0.05 were generated by Wilcoxon Rank-Sum test.

(Figure S3 continued)


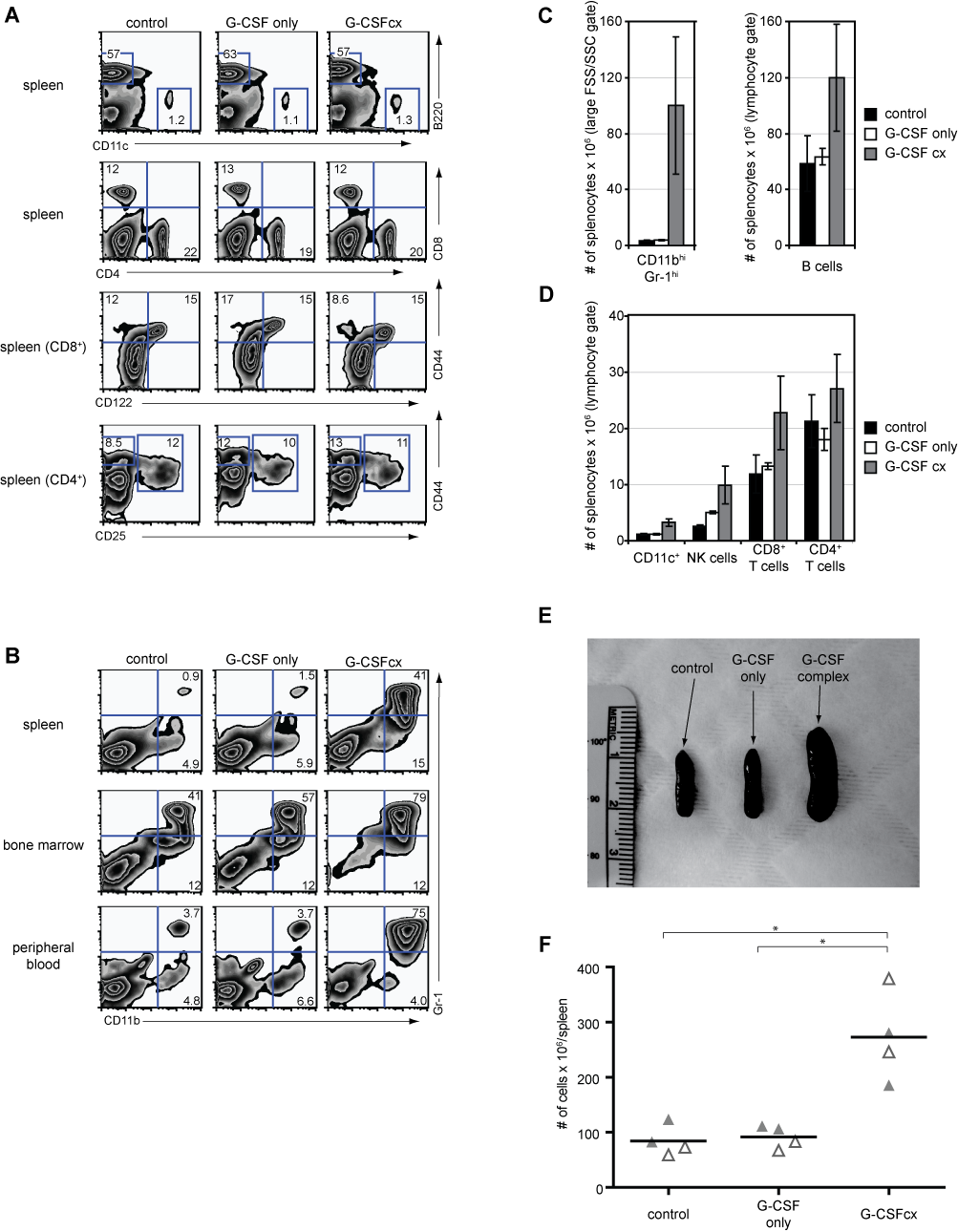


(Figure S4, continued on next page)

Figure S4. Long-term administration of G-CSF/anti-G-CSF mAb complexes induces splenomegaly and dramatic expansion of CD11b^+^Gr-1^+^ myeloid cells. B6 mice (n=2/group) were injected every 48 hours days with G-CSF/anti-G-CSF mAb complexes (1.5µg G-CSF plus 7.5µg anti-G-CSF mAb), cytokine alone (1.5µg G-CSF), or vehicle alone. On day 20 after the initiation of cytokine administration, spleen, bone marrow, and peripheral blood were harvested. (A) Spleens were stained for B cells, dendritic cells, and T cells, as indicated, and gated on a small lymphocyte gate. (B) As in ‘A’, except spleen, bone marrow, and peripheral blood were stained, as indicated, and gated on a large FSC/SSC gate. (C & D) The mean absolute numbers of cells as gated in ‘A & B’ for 2 mice per group. The error bars indicate the range of the two data points per mean. (E) Representative spleens from ‘A’. (F) The number of spleen cells per mouse from the experiment shown in ‘A-E’ (filled triangles) and a duplicate experiment (open triangles). The bar indicates the average. *P-values ≤ 0.05 were generated by Wilcoxon Rank-Sum test. For ‘A-E’, all data are representative of 2 independent experiments which are summarized in ‘F’.

(Figure S4 continued)


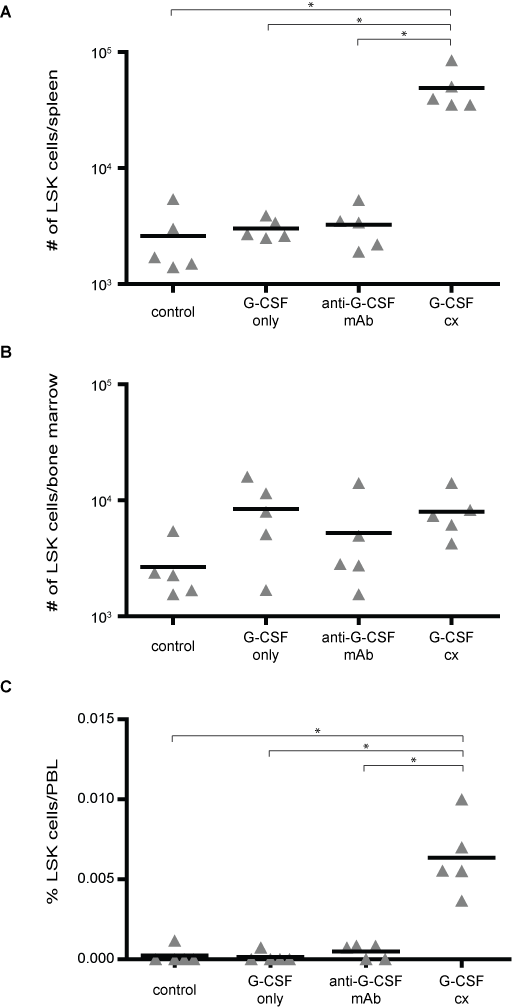


Figure S5. Administration of G-CSF/anti-G-CSF mAb complexes induces increased numbers of hematopoietic progenitor cells in the spleen and peripheral blood. B6 mice were injected every 48 hours days with G-CSF/anti-G-CSF mAb complexes (1.5µg G-CSF plus 7.5µg anti-G-CSF mAb), cytokine alone (1.5µg G-CSF), antibody alone (7.5µg G-CSF) or vehicle alone. On day 7 after the initiation of cytokine administration, (A) spleen, (B) bone marrow, and (C) peripheral blood were harvested. The absolute number Sca1^+^ckit^+^ (and lineage negative (B220^-^CD3^-^CD11b-NK1.1^-^Ter119^-^) cells was determined. The triangles indicate individual mice and the bar indicates the average per group. *P-values ≤ 0.05 were generated by Wilcoxon Rank-Sum test.

(Figure S5 continued)


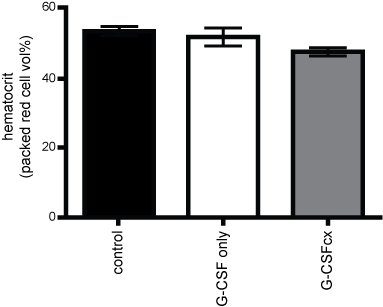


Figure S6. Extended administration of G-CSF/anti-G-CSF mAb complexes does not affect hematocrit. Packed cell volume was determined from animals treated with vehicle, G-CSF only, or G-CSF complex for 20 days as described in Figure S2. Peripheral blood centrifuged in capillary tubes before measuring red cell packed volume (excluding buffy coat) as a percent of total blood volume.


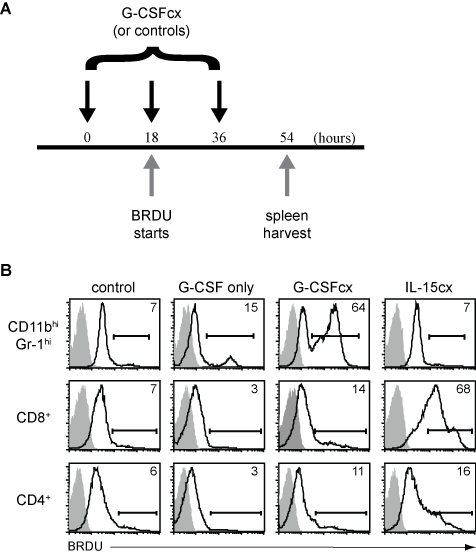


Figure S7. G-CSF/anti-G-CSF mAb complexes induce the proliferation of CD11b^+^Gr-1^+^ myeloid cells. (A) B6 mice were injected i.v. (at 0hr, 18hr, and 36hr) with G-CSF/anti-G-CSF mAb complexes (1.5µg G-CSF plus 7.5µg anti-G-CSF mAb), G-CSF alone (1.5µg G-CSF), vehicle alone, or IL-15/sIL-15Rα complexes (1.5µg G-CSF plus 7µg sIL-15Rα-Fc). At 18 hours, mice were also injected once i.v. with BrdU and placed on BrdU-containing drinking water. (B) At 54 hours, spleens were harvested and stained as indicated.


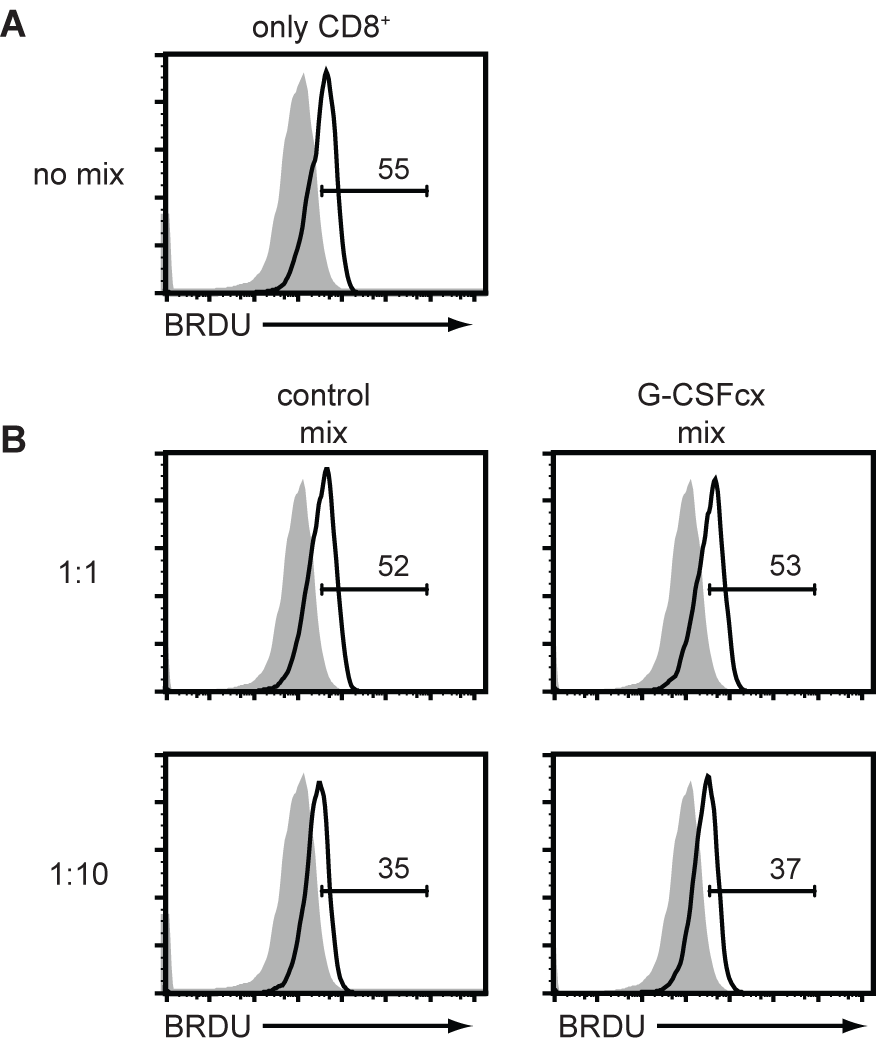


Figure S8. Activated CD8^+^ T cells proliferate normally when mixed with splenocytes from G-CSF/anti-G-CSF mAb complex-treated mice. B6 mice were injected with or without G-CSF/anti-G-CSF mAb complexes (1.5µg G-CSF plus 7.5µg anti-G-CSF mAb) on days 0, 2, and 4. On day 6, spleens were harvested and tested for suppressive ability on the proliferation of activated CD8^+^ T cells. For generation of activated CD8^+^ T cells, pmel-1 T cells were stimulated for 72 hours with hGP100 peptide (1μg/ml) and IL-12 (10ng/ml). Co-cultures of activated T cells with or without splenocytes (at a 1:1 or 1:10 ratio) were incubated overnight with (black line) or without (shaded) mIL-2 at 200ng/ml.

After overnight culture, cells were pulsed for 2 hours with BRDU and stained for flow cytometric analysis. The histograms show co-cultures of activated CD8^+^ T cells (A) without mixed splenocytes or (B) with splenocytes mixed at a 1:1 or 10:1 mixture.


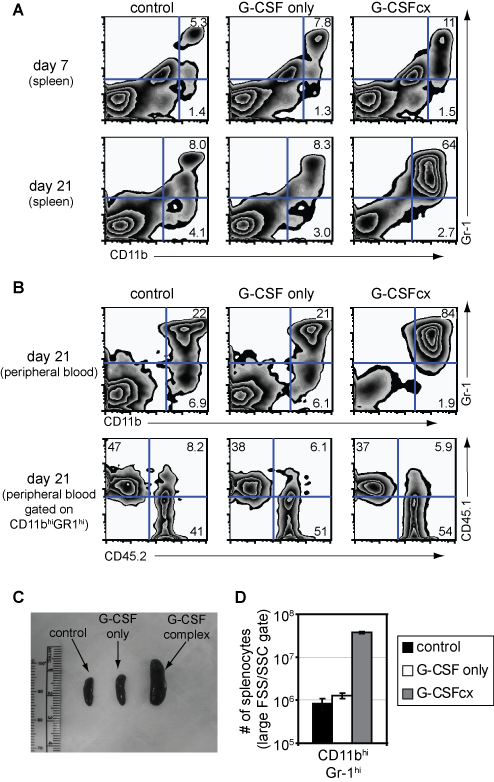


Figure S9. G-CSF/anti-G-CSF mAb complexes expand CD11b^+^Gr-1^+^ myeloid cells after bone marrow transplantation. (A) B6 mice were lethally irradiated on day -1 and then 2x10^6^ T- and B- depleted CD45.1 congenic bone marrow cells were transferred i.v. on day 0. Beginning one day later, and until sacrifice, mice received injections i.p. every 48 hours with G-CSF/anti-G-CSF mAb complexes (0.5µg G-CSF plus 2.5µg anti-G-CSF mAb), G-CSF alone (0.5µg G-CSF), or vehicle alone (n=2/group). Spleen cells were harvested on days 7 and 21, and the percentage of myeloid cells (CD11b^+^Gr-1^+^) was assessed based on a large FSS/SSC gate. (B) As in ‘A” except the percentage of myeloid cells (CD11b^+^Gr-1^+^) in the peripheral blood was assessed (upper row). The percentage of donor (CD45.1^+^) and recipient (CD45.2^+^) myeloid cells from peripheral blood was also determined (bottom row). (C) Picture of representative spleens from mice on day 21 after treatment as described in ‘A’. (D) As in ‘C’, except the absolute numbers of myeloid cells (CD11b^+^Gr-1^+^) was assessed.

(Figure S9 continued)


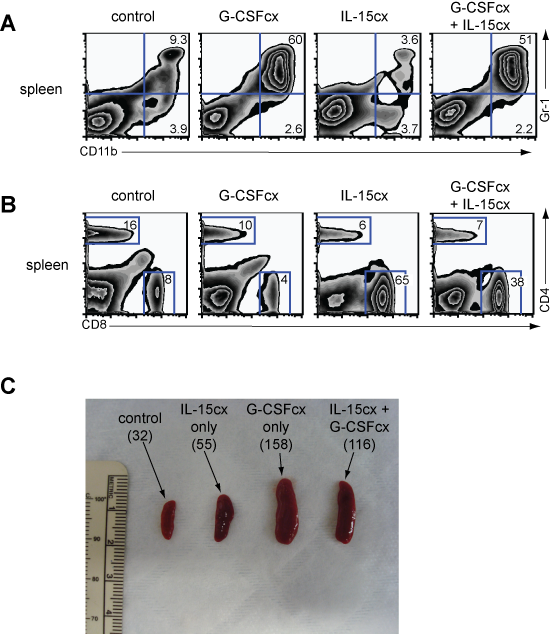


Figure S10. The combination of G-CSF/anti-G-CSF mAb complexes and IL-15/sIL-15Rα-Fc complexes induces more effective hematopoietic recovery following bone marrow transplantation. (A) B6 mice were lethally irradiated and 2x10^6^ T- and B- depleted CD45.1 congenic bone marrow cells were transferred i.v. one day later on day 0. Beginning one day later, mice received injections i.p. every 48 hours with G-CSF/anti-G-CSF mAb complexes (0.5µg G-CSF plus 2.5µg anti-G-CSF mAb), IL-15/sIL-15Rα complexes (1.5µg G-CSF plus 7µg sIL-15Rα-Fc), both complexes together, or vehicle alone. Spleen cells were harvested on day 21, and analyzed as indicated by FACS analysis. (B) As in ‘A’, except cells were gated with a tight lymphocyte gate. (C) Spleens in ‘A’ were photographed. The absolute number of cells recovered is indicated in parenthesis (x 10^6^).
